# Effect of Daily Oral *Lactobacillus plantarum* PS128 on Exercise Capacity Recovery after a Half-Marathon

**DOI:** 10.3390/nu13114023

**Published:** 2021-11-11

**Authors:** Szu-Kai Fu, Wei-Chin Tseng, Kuo-Wei Tseng, Chang-Chi Lai, Ying-Chieh Tsai, Hsia-Ling Tai, Chia-Chen Hsu

**Affiliations:** 1 Graduate Institute of Sports Training, College of Kinesiology, University of Taipei, Taipei 11153, Taiwan; skyfu218@gmail.com (S.-K.F.); danatai1008@gmail.com (H.-L.T.); 2 Department of Exercise and Health Sciences, College of Kinesiology, University of Taipei, Taipei 11153, Taiwan; speedceng@gmail.com (W.-C.T.); fossil0405@yahoo.com.tw (K.-W.T.); sports_injury0406@yahoo.com.tw (C.-C.L.); 3 Institute of Biochemistry and Molecular Biology, College of Life Sciences, National Yang-Ming Chiao-Tung University, Taipei 112304, Taiwan; tsaiyc@ym.edu.tw; 4 Department of Otorhinolaryngology, Taipei City Hospital, Taipei 10341, Taiwan

**Keywords:** *L. plantarum* PS128, muscle damage, exercise capacity recovery, half-marathon, gastrointestinal microbiota

## Abstract

A half-marathon (HM) is a vigorous high-intensity exercise, which could induce lower extremity musculoskeletal injury risks for recreational runners. They usually consume nonsteroidal anti-inflammatory drugs (NSAIDs) in order to shorten their return to play but ignore the side effects, such as peptic ulcers and renal and vascular disorders. *Lactobacillus plantarum* PS128 (PS128) could improve inflammation and oxidative stress by modulating the gut microbiota, thus potentially improving muscle damage and recovery. However, few studies have addressed the PS128 exercise capacity recovery 96 h after HM. Thus, this study aimed to investigate the effect of PS128 on exercise capacity and physiological adaptation after HM. A double-blind, randomized, placebo-controlled, counterbalanced, crossover trial was used for the experiment. HM was conducted at the beginning and end of the 4-week nutritional supplement administration. Eight recreational runners took two capsules (3 × 10^10^ CFU/capsule) of PS128 each morning and evening before meals for 4 weeks as the PS128 treatment (LT), or they took two capsules of placebo for 4 weeks as the placebo treatment (PT). In both treatments, an exercise capacity test (lower extremity muscle strength, anaerobic power, lower extremity explosive force, and aerobic capacity) and blood test (muscle fatigue, muscle damage, oxidative stress, and renal injury) were performed before the administration of the nutritional supplement (baseline), 48 h before HM (pre), and 0 h (0 h post), 3 h (3 h post), 24 h (24 h post), 48 h (48 h post), 72 h (72 h post), and 96 h (96 h post) after HM. There was no significant difference in the total duration of HM between PT and LT, but PT was found to be significantly higher than LT at Stage 4 (15,751–21,000 m) of HM (3394 ± 727 s vs. 2778 ± 551 s, *p* = 0.02). The lower extremity muscle strength measured using an isokinetic dynamometer in PT was significantly lower than that in LT at 72 h after HM. The lower extremity explosive force from the countermovement jump (CMJ) in PT was significantly decreased compared to 24 h prior. There was no significant difference between anaerobic power and aerobic capacity between the two treatments after HM. After HM, LT had lower muscle damage indices, such as myoglobin (3 h post-PT vs. -LT: 190.6 ± 118 ng/mL vs. 91.7 ± 68.6 ng/mL, *p* < 0.0001) and creatine phosphokinase (24 h post-PT vs. -LT: 875.8 ± 572.3 IU/L vs. 401 ± 295.7 IU/L, *p* < 0.0001). Blood urea nitrogen recovered in 24 h (24 h pre- vs. post-LT, *p* > 0.05) and higher superoxide dismutase was found in LT (96 h post-PT vs. -LT: 0.267 ± 0.088 U/mL vs. 0.462 ± 0.122 U/mL, *p* < 0.0001). In conclusion, PS128 supplementation was associated with an improvement in muscle damage, renal damage, and oxidative stress caused by HM through microbiota modulation and related metabolites but not in exercise capacity.

## 1. Introduction

Running is a popular sporting activity that can provide physical and psychological benefits to runners. However, long-distance running could induce lower extremity musculoskeletal injury risks. Prolonged running has been reported to contribute to muscular strength decline, increased creatine phosphokinase (CPK), delayed onset muscle soreness (DOMS), fatigue [1,2], and acute or chronic imbalance in multiple aspects of the human body [3].

Most muscle damage induced by exercise is due to increased oxidative stress, which results in a decrease in skeletal function [4]. Vigorous long-distance running also increases oxygen consumption, ischemia-reperfusion injury, white blood cell activation, inflammation, and reactive oxygen species (ROS) production [5,6].

Overuse of the lower extremity could lead to microtrauma [7], which triggers inflammatory responses, and the accumulative effects could lead to muscle injury and dysfunction [8]. Therefore, many athletes in endurance sports consume nonsteroidal anti-inflammatory drugs (NSAIDs) [9]. Although NSAIDs have anti-inflammatory and analgesic effects, they may cause side effects, such as peptic ulcers and renal and vascular disorders [10]. 

Therefore, it is necessary to find a strategy to reduce muscle damage and promote recovery with fewer side effects for runners. As defined by the Food and Agriculture Organization of the United Nations and World Health Organization, probiotics are “live microorganisms which when administered in adequate amounts confer a health benefit on the host” [11]. Probiotic strains with demonstrated health benefits belong primarily to the following genera: *Lactobacillus*, *Bifidobacterium*, *Saccharomyces*, *Enterococcus*, *Streptococcus*, *Pediococcus*, *Leuconostoc*, *Bacillus,* and *Escherichia* [12]. Probiotics have been shown to have strain-specific effects; for example, *L. casei* may improve the symptoms associated with rheumatoid arthritis [13], *Bifidobacterium bifidum* may reduce acute diarrhea and shorten the duration of hospitalization due to acute diarrhea in children [14], and *Saccharomyces boulardi* is effective in the treatment of acute gastroenteritis in children [15]. Therefore, the identification of specific strains is crucial.

Gastrointestinal health could be critical for the regulation of exercise adaptation to deal with diarrhea, nausea, and abdominal cramps during prolonged and exhaustive exercise. Probiotic supplements have been shown to improve the frequency, severity, and duration of respiratory infections and gastrointestinal diseases, which may be achieved through the interaction between the gut microbiota and the immune system [16]. In 2016, Jäger et al. administered two probiotic strains (Bifidobacterium breve BR03 and Streptococcus thermophilus FP4) to 16 healthy men who habitually performed resistance training for 21 days, and theyconducted eccentric contraction of the elbow flexor muscle injury intervention. It was found that the average maximum torque of isometric contraction between IL-6 and the elbow flexor was better than that of the placebo group within 72 h after the intervention of the muscle injury [17]. Some studies on probiotics have demonstrated the benefits of lactobacilli in sports science. *L. plantarum* TWK10 has been found to improve exercise capacity by restoring energy and facilitating muscle adaption in both clinical and preclinical studies [18,19]. *L.* helveticus *lafti* L10 has been demonstrated to improve mucosal immune response in elite athletes in addition to alleviating upper respiratory tract infection [13]. *Lactococcus lactis* JCM 5805 has been proven to alleviate upper respiratory tract infection without affecting the biomarkers of muscle damage and oxidative stress [20]. The two probiotic strains, L. rhamnosus IMC 501 and L. paracasei IMC 502, can improve the oxidative stress caused by the 4-week intensive training intervention [21]. Recreationally trained males consumed 20 g of casein plus 1 billion CFU B. coagulans GBI-30, 6086 daily for 2 weeks, resulting in reduced exercise-induced muscle damage and increased recovery [22]. It could enhance the adaptation rate to training and subsequently lead to a faster increase in hypertrophy and performance.

*Lactobacillus* is the predominant genus, which has >100 species and is rich in carbohydrates. Most lactobacilli are isolated from the gastrointestinal tract of humans and animals, as well as from vegetables and fermented foods. The members are Gram-positive, nonmotile, non-spore-forming, and acid-tolerant facultative anaerobes [23]. *Lactobacillus* sp. is regarded as a probiotic due to its health benefits for the human body, as demonstrated in several studies, for example, the improvement in type 2 diabetes mellitus [24], blood pressure [25], as well as anti-inflammatory and antioxidant effects [26,27]. *Lactobacillus plantarum* PS128 (PS128) isolated from *fu-tsai*, a traditional Taiwanese fermented vegetable food product [28], has been found to improve behavioral performance in patients with autism spectrum disorder [29]; it also exhibits anti-inflammatory and immune system regulatory effects [30] and helps control neuropsychiatric diseases by decreasing anxiety-like behaviors [31]. In recent studies, PS128 has been shown to be effective in decreasing the level of muscle damage, oxidative stress, and inflammatory symptoms caused by long-term exercise in endurance athletes [32]. PS128 supplementation modulated the athlete’s microbiota with significant decreases in Anaerotruncus, Caproiciproducens, Coprobacillus, Desulfovibrio, Dielma, Family_XIII, Holdemania, and Oxalobacter and increases in Akkermansia, Bifidobacterium, Butyricimonas, and Lactobacillus, and they showed more diversity [33]. Short-chain fatty acids (SCFAs) increased after the administration of PS128, which might be a result of the modulation of the associated microbiota.

However, there has been no study investigating whether long-term PS128 consumption in recreational runners decreases the level of muscle damage and promotes the recovery of exercise capacity after HM. Therefore, in this study, we evaluated the recovery of exercise capacity and the level of muscle damage in recreational runners with placebo and PS128 intake after HM.

## 2. Materials and Methods

### 2.1. Lactic Acid Bacteria

*Lactobacillus plantarum* PS128 was stored at the DSMZ German Collection of Microorganisms and Cell Cultures with the accession number DSM 28632. It was manufactured by Bened Biomedical Co., Ltd. (Taipei City, Taiwan) in the dosage form of capsules containing milky white powder. Each capsule weighed 425 ± 25 mg and contained 3 × 10^10^ CFU of PS128 in the shell of the microcrystalline cellulose. The product was stored at 4–8 °C. The placebo treatment (PT) received the same type of capsules filled with 425 ± 25 mg microcrystalline cellulose.

### 2.2. Participants

Eight recreational runners (four males: age: 27.0 ± 2.3 years; height: 176.8 ± 2.4 cm; weight: 77.7 ± 8.5 kg; BMI: 24.8 ± 2.4 kg/m2 and four females: age: 24.0 ± 0.8 years; height: 162.0 ± 4.3 cm; weight: 55.5 ± 1.0 kg; BMI: 21.2 ± 0.8 kg/m2) were included in the study. The participants had not undergone regular resistance training, aerobic exercise, or flexibility training in the past year; did not carry heavy things frequently; and did not have musculoskeletal injury. They were requested to maintain a normal daily routine within 48 h before trial-related activity and were prohibited from strenuous exercise, smoking, alcoholic beverage intake, or staying up late. During the use of PS128, the participants were prohibited from consuming other probiotics, such as fermented products (e.g., Yakult and yogurt), vitamins, minerals, Chinese medicine, and antibiotics. Participants who succeeded in the initial screening were informed of the study objectives, procedures, and potential risks, and written informed consent was obtained from them. The study was conducted according to the Declaration of Helsinki after obtaining approval from the institutional review board of the University of Taipei (approval no.: Taipei, Taiwan; no. IRB-2020-054).

### 2.3. Experimental Design

A double-blind, randomized, placebo-controlled, counterbalanced, crossover trial was used for the experiment. The period of nutritional supplementation was 4 weeks, in accordance with previous studies [21,32]. HM was conducted at the end of the 4-week nutritional supplementation. Participants took 2 capsules (3 × 10^10^ CFU/capsule) of PS128 each morning and evening before meals for 4 weeks as the PS128 treatment (LT, *n* = 8), or they took 2 capsules of placebo for 4 weeks as the PT (*n* = 8) in the control. After HM, an exercise capacity test, a blood test, and a 3-month washout period were conducted. The nutritional supplements taken by LT and PT were exchanged and taken for 4 weeks, and HM, an exercise capacity test, and a blood test were performed again. 

**Figure 1:**
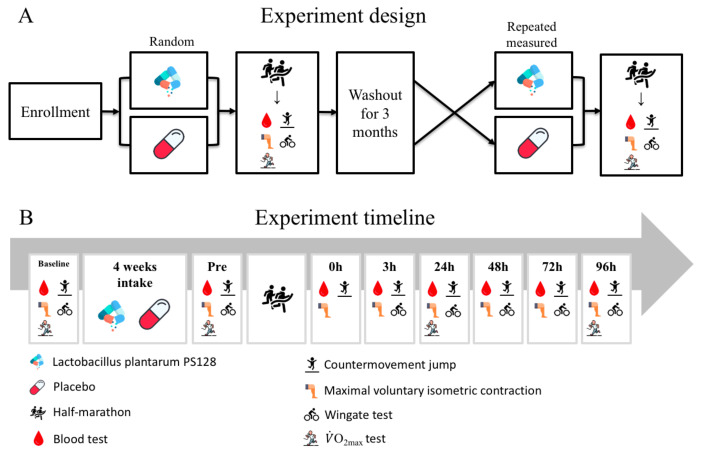
Experimental design flowchart. Experiment design (**A**): A double-blind, randomized, placebo-controlled, counterbalanced, crossover trial was used for the experiment. The period of nutritional supplementation was 4 weeks. Half-marathon (HM) was conducted at the end of the 4-week nutritional supplementation. Participants took 2 capsules of *Lactobacillus plantarum* PS128 (PS128) each morning and evening before meals for 4 weeks as the PS128 treatment (LT, *n* = 8), or they took 2 capsules of placebo for 4 weeks as the placebo treatment (PT, *n* = 8) in the control. After HM, an exercise capacity test, a blood test, and a 3-month washout period were conducted. Then, the two treatments were exchanged, and subsequent interventions and detection were performed. Experiment timeline (**B**): Baseline, pre, 0 h, 3 h, 24 h, 48 h, 72 h, and 96 h indicate the stage before the administration of the nutritional supplement, 48 h before the half-marathon intervention, immediately after the half-marathon intervention, 3 h after the half-marathon intervention, 24 h after the half-marathon intervention, 48 h after the half-marathon intervention, 72 h after the half-marathon intervention and 96 h after the half-marathon intervention, respectively.

The muscle damage caused by EIMD could last for approximately 96 h. Thus, in order to observe the period of return to play, in both treatments, an exercise capacity test and blood sampling were performed before nutritional supplement administration (baseline), 48 h before HM (pre), and 0 h (0 h post), 3 h (3 h post), 24 h (24 h post), 48 h (48 h post), 72 h (72 h post), and 96 h (96 h post) after HM (Figure 1). The exercise capacity assessment included lower extremity muscle strength, anaerobic power (except for 0 h post), lower extremity explosive force, and aerobic capacity (only at pre, 24 h post, and 96 h post) tests. Blood samples were used to analyze the biomarkers for muscle fatigue, muscle damage, oxidative stress, and renal injury.

### 2.4. Half-Marathon

At the Tianmu campus of the University of Taipei, equipped with a standard 400 m track and field stadium made of polyurethane (PU), all participants jogged for 5 min and carried out a 15 min dynamic warm-up before HM at 5 am. The total distance was 21 km, and the time was recoded every 5 km and 250 m, for a total of 4 time divisions (Stage 1: 0–5250 m; Stage 2: 5251–10,500 m; Stage 3: 10,501–15,750 m; Stage 4: 15,751–21,000 m). A station was placed at the 50 m point from the starting line to provide water and first-aid services, where emergency medical technicians were stationed to monitor the whole course.

### 2.5. Lower Extremity Muscle Strength Test

For lower extremity muscle strength testing, an isokinetic dynamometer (Biodex System Pro 4; Biodex Medical Systems, Shirley, NY, USA) was used to measure the maximal voluntary isometric contraction (MVIC) of knee extensors and flexors. Participants were familiarized with MVIC test procedures 2 weeks before their test. At a sitting position (hip flexion 85°), with knee flexion 30° over the dominant side and the shoulders, chest, hip, and thigh over the nondominant side fixed with straps, a participant was asked to perform knee extensor MVICs for 3 s with the dominant leg (defined as the leg the participant chooses to kick a ball with) (Montgomery and Shultz 2010) at the maximal exertion. The test was repeated thrice, with a 1 min interval between the two attempts. A knee flexors test was conducted, 5 min after the knee extensors test, at a sitting position (hip flexion 85°), with a knee flexion of 60° over the dominant side. Participants were encouraged and instructed orally by the study staff during their tests to ensure MVIC was achieved in each contraction [34]. Test results were analyzed by the Biodex System Pro 4 Curve Analysis software. Lower extremity muscle strength was presented as the peak torque per kg of body weight (Nm), and the maximum value among the three attempts was considered.

### 2.6. Anaerobic Power Test

The 30 s Wingate Anaerobic Test was conducted to determine the anaerobic power (W.kg^−1^), such as anaerobic peak power, anaerobic mean power, and fatigue index [35]. A bicycle dynamometer, Cyclus 2 (Avantronics Cyclus 2, Leipzig, Germany), was used, and the resistance coefficient was adjusted according to the sex and weight of the participants (male: weight × 0.8; female: weight × 0.77). The bicycle dynamometer seat was set at the most appropriate height where a participant’s knee joint could be kept at 30° flexion when the pedal of the same side was set at the lowest position. Participants were asked to pedal at 60 revolutions per minute (rpm) without resistance for 2 min before the test started. The study staff counted down from 10 s before the initiation of the actual test to alert the participants. During the actual test, the study staff encouraged the participants to reach their highest possible rpm.

### 2.7. Lower Extremity Explosive Force Test

The countermovement jump (CMJ) was used to test the explosive force of the lower extremities of the participants, and the values were defined by the height of the jump they could perform. A BTS force plate (P6000, BTS Bioengineering, Milano, Italy), fixed on the floor, was used for this test. A computer was connected to the force plate to collect the data. The jump height was calculated by introducing the flight time into the jump height formula using Microsoft Excel 2016 [36].

### 2.8. Aerobic Capacity Test

The Bruce maximal aerobic capacity test was conducted; the participants ran on a high-speed treadmill (H/P COSMOS Pulsar 3P, Nussdorf-Traunstein, Germany) [37] at pre, 24 h post, and 96 h post stages. The values were presented as the maximum oxygen consumption, measured by a cardiopulmonary exercise tester (Cosmed Quark CPET system; Rome, Italy). The test was initiated once a participant on the treadmill showed a sign of stable heart beats. The speed and slope started at 2.7 km/h and 10% and then were adjusted every 3 min according to the following settings: 4.0 km/h and 12%, 5.5 km/h and 14%, 6.8 km/h and 16%, 8.0 km/h and 18%, 8.9 km/h and 20%, 9.7 km/h and 22%, and 10.5 km/h and 24% (end of test). To ensure that participants exerted their maximal effort, the test results should meet two of the following three criteria: (1) voluntary request to stop the test, (2) respiratory exchange ratio of >1.15, and (3) oxygen consumption change of ≤2 mL·kg^−1^·min^−1^ from a lower-intensity to higher-intensity test level [38].

### 2.9. Blood Sampling

Blood samples were collected from the hand veins of the upper extremities at baseline, pre, 0 h post, 3 h post, 24 h post, 48 h post, 72 h post, and 96 h post stages to analyze muscle fatigue indicators, such as branched-chain amino acids (BCAA) and ammonia (NH_3_); muscle damage indicators, such as myoglobin, lactate dehydrogenase (LDH), and creatinine phosphokinase (CPK); renal injury indicators, such as blood urea nitrogen (BUN); and oxidative stress indicators, such as superoxide dismutase (SOD) and catalase (CAT). The blood samples were collected in tubes and centrifuged to obtain plasma and serum. Collection tubes containing anticoagulant heparin were used to obtain plasma, and those without anticoagulants were used to obtain serum. The plasma and serum were stored at −70 °C for further analysis.

### 2.10. Biochemical Variables

NH_3_, LDH, CPK, and BUN in the serum were detected using a Beckman Coulter UniCel DxC 800 Automatic Biochemical Analyzer (Beckman Coulter, Fullerton, CA, USA). Myoglobin was detected using a Beckman Coulter UniCel Dxl 810 Automatic Biochemical Analyzer (Beckman Coulter, Fullerton, CA, USA). The activities of SOD and CAT were assayed in the serum samples by using commercial kits (SOD: Cayman Chemicals Inc., 706002, Ann Arbour, MI, USA; CAT: Cayman Chemicals Inc., 707002, Ann Arbour, MI, USA), following the manufacturer’s instructions. The activities of SOD and CAT were calculated using an equation obtained from the linear regression of the standard curve. BCAA in the serum were quantified using a commercial colorimetric kit (BioVision, K564-100, Milpitas, CA, USA), following the manufacturer’s instructions. All samples and standards were measured in duplicate.

### 2.11. Statistical Analysis

The mean ± SD was used to describe all dependent variables. All statistical tests were conducted using GraphPad Prism (Version 8; GraphPad Software, San Diego, CA, USA) and Excel 2016, with statistical significance set at *p* ≤ 0.05. The normality assumption for each variable was verified using the Shapiro–Wilk test, which confirmed the normal distribution of data. A prior evaluation of the homogeneity of variance and sphericity was conducted using Levene’s test and Mauchly’s test, respectively. Consequently, a mixed analysis of variance (ANOVA) was applied to examine the changes in each variable over time between the two treatments, considering the within-subject factor time (pre, 0 h post, 3 h post, 24 h post, 48 h post, 72 h post, and 96 h post) and the between-subject factor group (LT and PT). Follow-up statistical tests included one-way repeated-measures ANOVA with Tukey’s post hoc test and pairwise comparisons with Sidak adjustments. To avoid individual variation, the measurements of exercise performance were normalized to the percentage of the premeasurement (posttest/pretest) × 100% for comparison. The results are shown as the percentage (%) ± standard deviation (%).

## 3. Results

### 3.1. Test of Homogeneity

The results of a test of homogeneity are shown in Table 1. Mean ± SD was used to describe all dependent variables. All statistical tests were conducted using Excel 2016 with statistical significance set at *p* ≤ 0.05. The results of the *t*-test show that there was no significant difference between the PT and LT at baseline.

Comparing the exercise capacity and blood test from pre-PT and pre-LT, Mean ± SD was used to describe all dependent variables. All statistical tests were conducted using Excel 2016 with statistical significance set at *p* ≤ 0.05. The results of the *t*-test showed that there was no significant difference between the two treatments in various indicators (Table 2).

### 3.2. Half-Marathon

**Figure 2:**
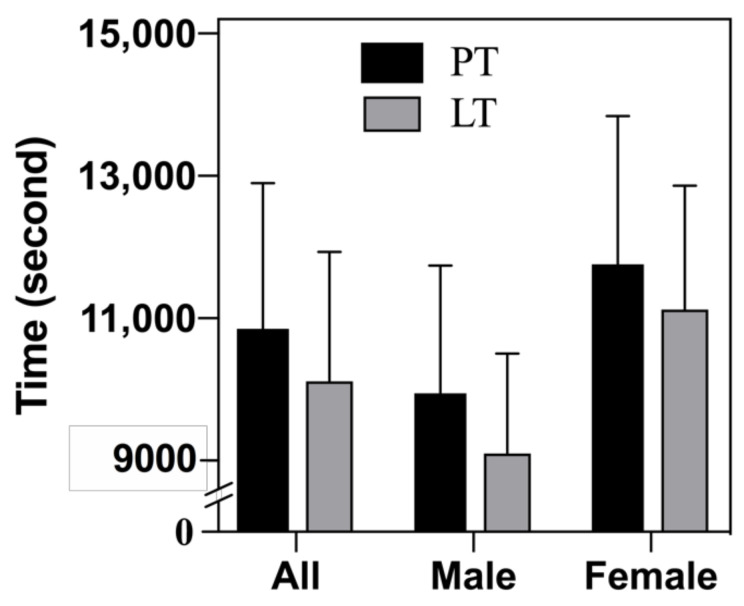
Comparison of the completion time of the half-marathon. The data are represented as mean ± standard deviation. Placebo treatment (PT); Lactobacillus plantarum PS128 treatment (LT). *p* < 0.05 was considered to be a statistically significant difference within and between the groups.

The environmental conditions during HM were as follows for PT (*n* = 4) and LT (*n* = 4): 17.6–18.1 °C (05:00 am.–09:00 am.), relative humidity of 80–86%, and wind speed of 1.1–1.2 m/s. After a 3-month washout and the exchange of nutritional supplements in the two treatments, the environmental conditions during HM were as follows for PT (*n* = 4) and LT (*n* = 4): 17.1–17.9 °C (05:00 am.−09:00 am.), relative humidity of 77–79%, and wind speed of 1.0–1.1 m/s. The total duration in which the eight PT participants completed HM varied from 02:01:37 to 04:08:00 (HH:MM:SS), with a mean duration of 03:00:52 ± 00:34:05 (02:45:44 ± 00:29:56 in males and 03:16:00 ± 00:34:42 in females). The total duration in which the eight LT participants completed HM varied from 01:57:09 to 03:46:58, with a mean duration of 02:46:34 ± 00:29:55 (02:46:34 ± 00:29:55 in males and 03:02:25 ± 00:29:47 in females). There was no significant difference between PT and LT (*p* = 0.819) (males undergoing PT and LT: *p* = 0.895; females undergoing PT and LT: *p* = 0.950; sex in PT: *p* = 0.316; sex in LT: *p* = 0.249 (Figure 2)).

**Figure 3:**
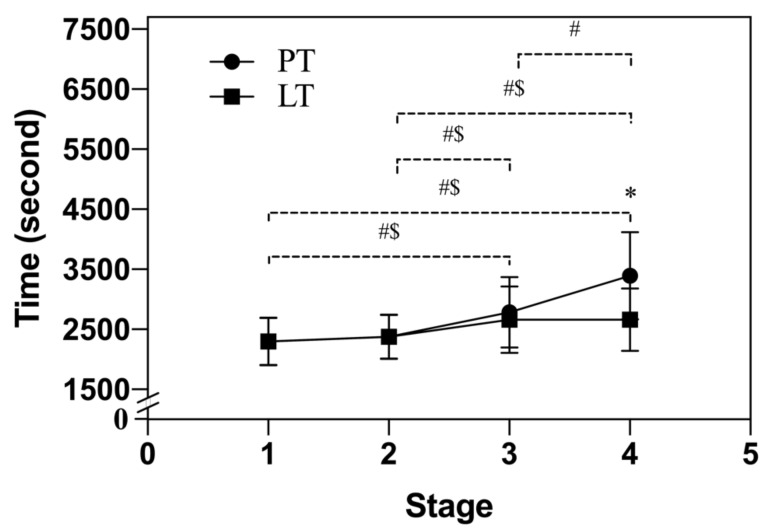
Comparison of the split time of the half-marathon. The data are represented as mean ± standard deviation. Placebo treatment (PT); Lactobacillus plantarum PS128 treatment (LT). Stage 1 distance is 0–5250 m; Stage 2 distance is 5251–10,500 m; Stage 3 distance is 10,501–15,750 m; Stage 4 distance is 15,751–21,000 m; * indicates a significant difference between PT and LT (*p* < 0.05); # indicates that PT has a significant difference in split time (*p* < 0.05); $ indicates that LT has a significant difference in split time (*p* < 0.05).

A two-way repeated-measures ANOVA detected that there was a significant interaction between time and stage (*p* < 0.0001) for the split time of HM. Therefore, the pairwise comparisons from Tukey multiple comparisons tests showed that the split times of HM were significantly different between Stage 1 and Stage 3 (*p* < 0.0001), Stage 1 and Stage 4 (*p* < 0.0001), Stage 2 and Stage 3 (*p* < 0.0001), Stage 2 and Stage 4 (*p* < 0.0001), and Stage 3 and Stage 4 (*p* < 0.0001). Sidak multiple comparisons tests showed that the split time of HM for PT was significantly lower than LT at Stage 4 (3394 ± 727 s vs. 2778 ± 551 s, *p* < 0.0001) (Figure 3).

### 3.3. Analysis of Lower Extremity Muscle Strength

**Figure 4:**
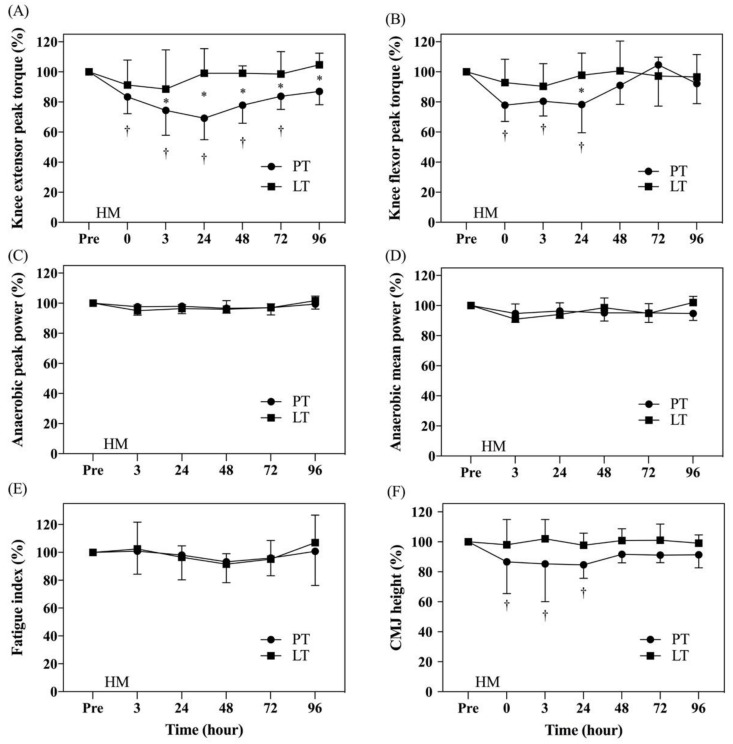
Comparison of normalized exercise capacity results. The data are represented as mean ± standard deviation. Knee extensor peak torque (**A**); knee flexor peak torque (**B**); anaerobic peak power (**C**); anaerobic mean power (**D**); fatigue index (**E**); countermovement jump (CMJ) height **(F**). HM: half-marathon; † represents that there was a significant change from pre to post time points in the placebo treatment (PT); * represents that there was a significant difference at the same time point between PT and LT. *p* < 0.05 was considered to be a statistically significant difference within and between the groups.

MVICs of knee extensors and flexors measured by an isokinetic dynamometer in lower extremity power test are shown in Figure 4A,B. A two-way repeated-measures ANOVA detected that there was a significant time by group interaction (*p* = 0.002) for knee extensor peak torque. Therefore, the pairwise comparisons from Tukey multiple comparisons tests showed that knee extensor peak torque decreased from the pre to 0 h post (*p* = 0.0136), 3 h post (*p* < 0.0001), 24 h post (*p* < 0.0001), 48 h post (*p* = 0.0004), and 72 h post (*p* = 0.017) stages in PT. The knee extensor peak torque in LT showed no significant difference after HM. Sidak multiple comparisons tests showed that the knee extensor peak torque for PT was significantly lower than that for LT at 3 h post (*p* = 0.025), 24 h post (*p* < 0.0001), 48 h post (*p* = 0.0003), 72 h post (*p* = 0.0173), and 96 h post (*p* = 0.0029) stages (Figure 4A).

A two-way repeated-measures ANOVA detected that there was a significant time by group interaction (*p* = 0.0394) for knee flexor peak torque. Therefore, the pairwise comparisons from Tukey multiple comparisons tests showed that knee flexor peak torque decreased from the pre to 0 h post (*p* = 0.0075), 3 h post (*p* = 0.0256), and 24 h post (*p* = 0.009) stages in PT. The knee flexor peak torque in LT showed no significant difference after HM. Sidak multiple comparisons tests showed that the knee flexor peak torque for PT was significantly lower than that for LT at the 24 h post stage (*p* = 0.0116) (Figure 4B). In sum, LT showed less decline in the strength of knee extensors and knee flexors after HM.

### 3.4. Analysis of Anaerobic Power

Anaerobic power was tested by a 30 s Wingate Anaerobic Test, and the results are shown in Figure 4C–E. No significant change in the anaerobic peak power (Figure 4C), mean power (Figure 4D), or fatigue index (Figure 4E) before and after HM was observed in PT or LT.

### 3.5. Analysis of Lower Extremity Muscle Explosive Force

Lower extremity explosive force was measured by CMJ height, as shown in Figure 4F. No significant difference in lower extremity explosive force before and after HM was observed between PT and LT. However, in PT, the lower extremity explosive force at 0 h post, 3 h post, and 24 h post stages was significantly lower than that at the pre stage (*p* < 0.05). In LT, the result at each time point after HM did not decrease (Figure 4F), indicating less decline in lower extremity explosive force.

### 3.6. Analysis of Aerobic Capacity and Body Composition

The Bruce maximal aerobic capacity was tested using a high-speed treadmill and presented as maximal oxygen consumption. There were no significant changes in maximal oxygen consumption after HM in PT and LT.

### 3.7. Analysis of Blood Samples

**Figure 5:**
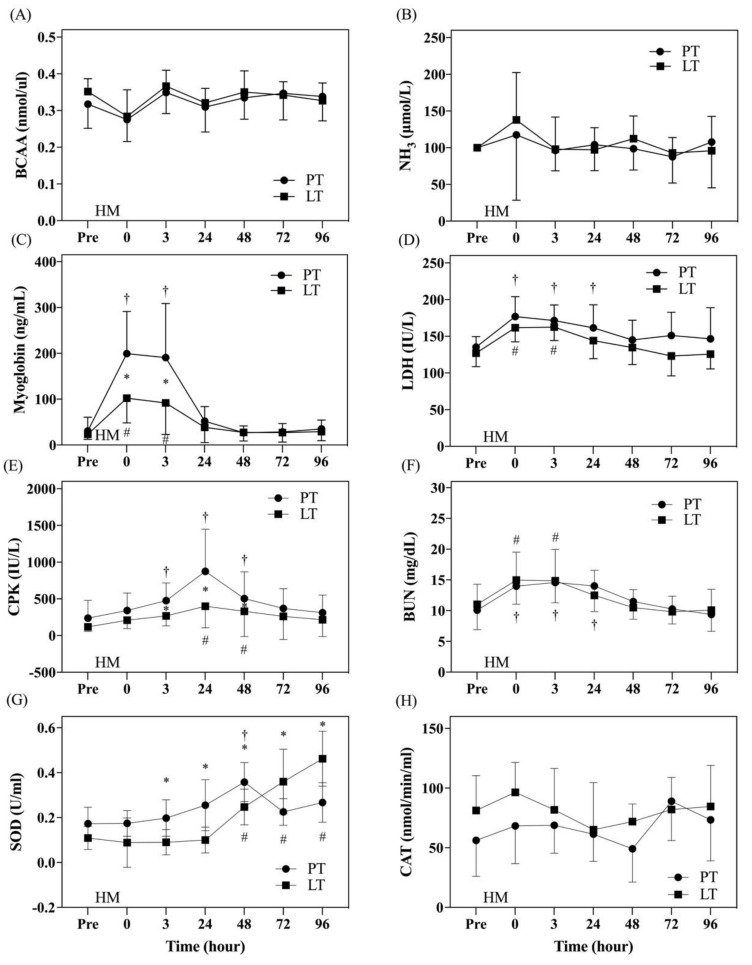
Comparison of blood tests. The data are represented as mean ± standard deviation. Branched-chain amino acid (BCAA) in Figure (**A**); blood ammonia level (NH_3_) in Figure (**B**); myoglobin in Figure (**C**); lactate dehydrogenase (LDH) in Figure (**D**); creatine phosphokinase (CPK) in Figure (**E**); blood urea nitrogen (BUN) in Figure (**F**); superoxide dismutase (SOD) in Figure (**G**); catalase (CAT) in Figure (**H**). ● represents the placebo treatment (PT); ■ represents the PS128 treatment (LT). HM: half-marathon; * represents that there was a significant difference at the same time point between PT and IG; † represents that there was a significant change from pre to post time points in PT; # represents that there was a significant change from pre to post time points in IG. *p* < 0.05 was considered to be a statistically significant difference within and between the groups.

There was no significant difference in the muscle fatigue factor after HM between PT and LT (Figure 5A,B). Muscle damage indicators were significantly elevated after HM in both treatments (Figure 5C–E). Myoglobin (0 h post and 3 h post; Figure 5C) and CPK (3 h post, 24 h post, and 48 h post; Figure 5E) in PT were significantly higher than those in LT, indicating apparently more serious muscle damage in PT. BUN, the indicator of renal injury, was significantly elevated after HM in both treatments, but it returned to baseline at 24 h post-LT (Figure 5F), indicating faster recovery. Although SOD, the indicator of antioxidation function, at 3 h post-, 24 h post-, and 48 h post-LT was significantly lower than that in PT (*p* < 0.05), it was significantly higher in LT than in PT at 72 h post and 96 h post stages (*p* < 0.05; Figure 5G), suggesting better antioxidation function in LT. There was no significant difference in CAT at any time point between the two treatments (*p* > 0.05; Figure 5H).

## 4. Discussion

There was no significant difference between PT and LT in HM, indicating that taking PS128 for 4 weeks did not significantly improve the performance of HM. However, comparing the split time of HM, it was found that only PT in Stage 4 was significantly slower than LT. However, there was no significant difference between Stage 3 and Stage 4 in LT. This showed that LT had a lower level of fatigue compared to PT.

In order to explore the difference in HM between the two treatments, the exercise capacity was tested; after 4 weeks of nutritional supplements, there were no significant differences in lower limb muscle strength, anaerobic power, lower limb explosive power, or aerobic capacity. From the analysis of blood tests, there were no significant differences in indicators such as muscle fatigue, muscle damage, renal damage, and antioxidant indicators. It could be seen that only taking PS128 for 4 weeks did not significantly improve exercise capacity. This showed that the mechanism of better anti-fatigue ability in LT during HM was not due to the improvement in exercise performance. This could be attributed to less muscle damage suffered; for example, myoglobin, LDH, and CPK were significantly lower in LT after HM compared with PT, which improved participants’ ability to maintain pace.

From the perspective of the recovery of exercise capacity after the HM intervention, it could be found that LT did not significantly decrease after the HM intervention, and it was significantly greater than PT at 3 h post, 24 h post, 48 h post, 72 h post, and 96 h post stages, indicating that it had better anti-fatigue ability or recovery. Long-distance running usually involves a large amount of quadriceps and eccentric contraction to absorb the impact of body weight when the heel comes into contact with the floor, causing muscle damage and leading to patella pain syndrome, iliotibial band syndrome, and patellar tendinitis. Such symptoms indirectly affect the maximum torque of the knee extensor muscles [39]. The 2004 study by Dowson et al. on eight male and four female recreational runners showed significant decreases in the knee extensor peak torque within 24 h after HM (return to baseline at 96 h post) and no significant change in knee flexor peak torque, which is consistent with the findings of this study [40]. Knee extensor peak torque is associated with the exercise capacity in the squat jump (SJ) and CMJ [41]. The study by Martínez-Sánchez et al. in 2017 on 21 male recreational runners showed significant decreases in both SJ and CMJ height and obvious muscle soreness up to 72 h after HM [42]. Similarly, in the study by Wiewelhove et al. in 2018, approximately 46 male recreational runners showed obvious decreases in CMJ height after HM [43]. The CMJ height in PT was significantly reduced after HM intervention and lasted for 24 h, but LT did not change at all. In the absence of a significant improvement in exercise capacity indicators, the maximum muscle strength and explosive power in LT after the HM intervention did not significantly decrease. This result highlights the need for further investigation of the mechanism of influence from changes in blood indicators.

Reduced muscle damage and faster recovery may lead to an enhanced adaptation rate to training and, subsequently, to a faster increase in hypertrophy and performance. Exercise-induced skeletal muscle tissue damage occurs as a result of the forced lengthening of an active muscle, which directly causes microtears of the myofibrils, resulting in muscle soreness and swelling, and decreased forced production. The initial reaction is followed by a secondary inflammatory response integral to skeletal muscle repair and recovery response. While muscle damage appears to be an important component of muscular adaptation to exercise, a reduction in and not a complete prevention of muscle damage, as shown by the addition of B. coagulans GBI-30 and 6086 to casein, might be optimal [22]. In blood analysis, muscle fatigue indices (e.g., BCAA and NH_3_) showed no significant difference in recovery after HM between the two treatments, whereas muscle damage indices (e.g., myoglobin and CPK) after HM in LT were significantly lower than those in PT, indicating the positive effect of the nutritional supplement on muscle damage. This is consistent with the findings of the study by Huang et al. [32], where the negative impact of high-intensity sports on exercise capacity was reduced by improving anti-inflammatory, antioxidation, and metabolic functions. High-intensity exercise may result in muscle damage, an imbalance of calcium homeostasis, the infiltration of neutrophils, and the production of free radicals and cytohormone metabolites, thereby resulting in elevated inflammatory (e.g., CPK and LDH) and oxidative stress-related (e.g., SOD and CAT) biomarkers [44]. DOMS is primarily caused by exercise-induced muscle damage (EIMD), frequently after unaccustomed or damaging exercise (e.g., downhill running). Leucocyte infiltration in injured tissues may cause muscular fiber damage and secondary inflammation [10,45], combined with the associated symptoms persisting for several days after exercise, such as muscle soreness; a reduced pain threshold; local swelling; a transient decrease in maximal power; and elevated levels of myoglobin, LDH, and CPK [46,47]. Similarly, in this study, muscle damage indices (i.e., myoglobin, LDH, and CPK) showed a significant increase, suggesting DOMS; lower extremity maximal muscle strength showed a significant decrease after HM in both PT and LT, as demonstrated in the study by Boccia et al. in 2017 [48]. No gender difference was observed in the study by Boccia et al. in 2018 [49]. However, after PS128 intervention, muscle damage indices were improved, the antioxidation indicator SOD elevated significantly, and exercise capacity was superior to that in PT, which seems to be the mechanism of action for PS128 to improve exercise capacity after HM, such as lower extremity muscle strength and lower extremity explosive force. PS128 has a similar mechanism to other nutritional supplements (i.e., fruit extracts, plant extracts, herb extracts, amino acids, and proteins) [50] in reducing EIMD, where muscle damage, DOMS, and inflammation are reduced and muscle function is improved via antioxidative and anti-inflammatory mechanisms. In terms of renal injury, BUN elevated significantly after HM in both LT and PT, as demonstrated by McCullough et al. [51]. It returned to baseline at 24 h post-LT, indicating better recovery. The mechanism that affects the changes in blood indicators needs to be explored based on the regulation mechanism of PS128 in the immune system.

PS128 reduced the production of lipopolysaccharide-induced proinflammatory cytokines in a RAW 264.7 mouse macrophage model and had positive regulatory effects on inflammation, oxidation, and metabolism caused by high-intensity exercise [32], as evidenced in this study, in which PS128 had significant regulatory effects on myoglobin, LDH, CPK, and SOD. In both clinical and nonclinical studies, *L. plantarum* has been demonstrated to exert anti-inflammatory activity and a regulatory effect on the immune response in hosts. Teichoic acids (TAs) produced by *L. plantarum* are the crucial substances that regulate the systematic immune response in mice [30]. By gene sequence alignment, it was found that *L. plantarum* PS128, WCFS1, and B21 have the core genes responsible for the metabolism, translation, and modification of TAs [30].

Despite the promising results, this study had several limitations. Although it used a repeated-measures design to include more participants (PT, *n* = 8; LT, *n* = 8), the low number of participants makes the results highly unreliable due to the low representation. The original aim was for 16 participants to complete the study. Recruitment was hindered by an unexpectedly high percentage of recreational runners who could not accommodate the experiment time of this study. As it was not possible to run on the road like in an actual competition, the test could only be carried out on a 400 m track in a field stadium, but the environmental factors were well controlled. The strengths of this study were that there were large number of sampling time points during the experimental period to investigate the exact reaction of related biochemical indicators with conditions of physiological limitation. This could obtain more information about the situation of recovery after HM. The detailed mechanisms affected by probiotics should be further investigated via systemic biological tools, such as proteomics and metabolomics, in the future.

## 5. Conclusions

In conclusion, PS128 supplementation was associated with an improvement in muscle damage, renal damage and oxidative stress caused by HM through microbiota modulation and related metabolites, but not in exercise capacity, thus neutralizing the ROS caused by exhaustive and prolonged exercise. Probiotics may represent an effective supplementation for athletes to establish an appropriate antioxidant barrier for preventing dangerous levels of oxidative stress. They could also shorten the return to play due to the consumption of PS128 instead of NSAIDs.

## Figures and Tables

**Table 1:** Test of homogeneity.

Exercise Capacity and Blood Samples	Baseline PT (*n* = 8)	Baseline LT (*n* = 8)	*p*-Value
Lower extremity muscle strength			
knee extensor peak torque (N-m/kg)	3.21 ± 0.88	3.08 ± 0.84	0.38
knee flexor peak torque (N-m/kg)	1.23 ± 0.35	1.15 ± 0.31	0.33
Anaerobic capacity			
anaerobic peak power (w/kg)	10.06 ± 1.66	10 ± 1.37	0.47
anaerobic mean power (w/kg)	7.05 ± 1.23	7.16 ± 1.47	0.44
fatigue index	14.95 ± 5.49	14.24 ± 3.32	0.38
Explosive force of the lower extremities			
CMJ height (cm)	32 ± 7.86	27.38 ± 11.49	0.18
Muscle fatigue			
BCAA (nmol/ul)	0.33 ± 0.07	0.34 ± 0.04	0.29
NH_3_ (μmol/L)	35.63 ± 20.56	28.25 ± 6.48	0.18
Muscle damage			
Myoglobin (ng/mL)	23.79 ± 9.65	29.72 ± 30.73	0.31
LDH (IU/L)	132.25 ± 8.92	132 ± 21.47	0.49
CPK (IU/L)	168.25 ± 125.41	188.13 ± 233.09	0.42
Renal injury			
BUN (mg/dL)	10.22 ± 3.14	10.79 ± 3.25	0.36
Anti-oxidative capacity			
SOD (U/mL)	0.11 ± 0.09	0.16 ± 0.06	0.10
CAT (nmol/min/mL)	64.88 ± 28.24	67.8 ± 24.9	0.41

The data are represented as mean ± standard deviation. Placebo treatment (PT); Lactobacillus plantarum PS128 treatment (LT); baseline is the time point before taking nutritional supplements; countermovement jump (CMJ); branched-chain amino acid (BCAA); blood ammonia (NH_3_); lactate dehydrogenase (LDH); creatine phosphokinase (CPK); blood urea nitrogen (BUN); superoxide dismutase (SOD); catalase (CAT). *p* < 0.05 was considered to be a statistically significant difference between the groups.

**Table 2:** Comparison of exercise capacity and blood test between PT and LT after taking nutritional supplements for 4 weeks.

	Pre-PT (*n* = 8)	Pre-LT (*n* = 8)	*p*-Value
Lower extremity muscle strength			
knee extensor peak torque (N-m/kg)	3.1 ± 0.82	3.07 ± 0.84	0.47
knee flexor peak torque (N-m/kg)	1.21 ± 0.36	1.14 ± 0.29	0.32
Anaerobic capacity			
anaerobic peak power (w/kg)	10.04 ± 1.6	10.07 ± 1.5	0.48
anaerobic mean power (w/kg)	7.06 ± 1.3	7.04 ± 1.54	0.49
fatigue index	14.88 ± 5.38	14.61 ± 3.91	0.46
Explosive force of the lower extremities			
CMJ height (cm)	31.86 ± 8.04	31.94 ± 8.07	0.49
Muscle fatigue			
BCAA (nmol/ul)	0.33 ± 0.07	0.33 ± 0.04	0.49
NH_3_ (μmol/L)	35.25 ± 20.81	27.63 ± 6.55	0.18
Muscle damage			
Myoglobin (ng/mL)	23.98 ± 10.04	29.76 ± 30.73	0.31
LDH (IU/L)	131.13 ± 10.53	131.25 ± 21.59	0.49
CPK (IU/L)	167.5 ± 125.62	187.38 ± 233.51	0.42
Renal injury			
BUN (mg/dL)	10.24 ± 3.18	10.86 ± 3.32	0.35
Anti-oxidative capacity			
SOD (U/mL)	0.13 ± 0.09	0.15 ± 0.05	0.29
CAT (nmol/min/mL)	62.48 ± 42.97	57.81 ± 33.9	0.41

The data are represented as mean ± standard deviation. Placebo treatment (PT); Lactobacillus plantarum PS128 treatment (LT); pre represents the time point of 48 h before the half-marathon intervention; countermovement jump (CMJ); branched-chain amino acid (BCAA); blood ammonia (NH_3_); lactate dehydrogenase (LDH); creatine phosphokinase (CPK); blood urea nitrogen (BUN); superoxide dismutase (SOD); catalase (CAT). *p* < 0.05 was considered to be a statistically significant difference between the groups.

## Data Availability

The data presented in this study are available on request from the corresponding author. The data are not publicly available due to privacy.
